# Molecular Epidemiology and Risk Factors of Ventilator-Associated Pneumonia Infection Caused by Carbapenem-Resistant Enterobacteriaceae

**DOI:** 10.3389/fphar.2019.00262

**Published:** 2019-03-22

**Authors:** Bo Gao, Xiandong Li, Fengmei Yang, Wei Chen, Ying Zhao, Gang Bai, Zhaoyong Zhang

**Affiliations:** ^1^Department of Laboratory Medicine, Taihe Hospital, Hubei University of Medicine, Shiyan, China; ^2^Department of Obstetrics and Gynecology, Taihe Hospital, Hubei University of Medicine, Shiyan, China; ^3^Department of Ultrasound, Taihe Hospital, Hubei University of Medicine, Shiyan, China

**Keywords:** ventilator-associated pneumonia, carbapenem-resistant Enterobacteriaceae, risk factor, resistance, gene

## Abstract

Ventilator-associated pneumonia (VAP) infection caused by carbapenem-resistant Enterobacteriaceae (CRE) is becoming more prevalent, thus seriously affecting patient outcomes. In this paper, we studied the drug resistance mechanism and epidemiological characteristics of CRE, and analyzed the infection and prognosis factors of VAP caused by CRE, to provide evidence for effective control of nosocomial infection in patients with VAP. A total of 58 non-repetitive CRE strains of VAP were collected from January 2016 to June 2018. To explore the risk factors of CRE infection, 1:2 group case control method was used to select non CRE infection patients at the same period as the control group. Among the 58 CRE strains, the most common isolates included *Klebsiella pneumoniae* and *Escherichia coli*. All strains were sensitive to polymyxin B, which features better sensitivity to other antibiotics such as minocycline, trimethoprim/sulfamethoxazole, and amikacin. Multiple drug resistance genes were detected at the same time in most strains. KPC-2 was the most common carbapenemase-resistant gene in *Klebsiella pneumoniae*, whereas NDM-1 was more common in *Escherichia coli*. The risk factors correlated with CRE infection included intensive care unit (ICU) occupancy time >7 days (OR = 2.793; 95% CI 1.439~5.421), antibiotic exposure during hospital stay including those to enzyme inhibitors (OR = 1.977; 95% CI 1.025~3.812), carbapenems (OR = 3.268; 95% CI 1.671~6.392), antibiotic combination therapy(OR = 1.951; 95% CI 1.020~3.732), and nerve damage (OR = 3.013; 95% CI 1.278~7.101). Multivariable analysis showed that ICU stay >7 days (OR = 1.867; 95% CI 1.609~20.026), beta-lactamase inhibitor antibiotics (OR = 7.750; 95% CI 2.219~27.071), and carbapenem (OR = 9.143; 95% CI 2.259~37.01) are independent risk factors for VAP carbapenem caused by Carbapenem-resistant Enterobacteriaceae. A high resistance rate of CRE isolated from VAP indicated that the infected patients featured higher mortality and longer hospital stay time than the control group. Multiple risk factors for CRE infection and their control can effectively prevent the spread of VAP.

## Introduction

Ventilator associated pneumonia (VAP) is a pulmonary parenchymal infection complicated by mechanical ventilation > 48 h. This condition is one of the common and severe complications during mechanical ventilation, and the main factor affecting patient prognosis (Rouzé et al., 2018). Enterobacteriaceae are the most common pathogenic bacteria causing VAP, among which carbapenem-resistant Enterobacteriaceae (CRE) bacteria have attracted attention due to their high resistance rate to most antibiotics.

High morbidity and mortality are closely related to the infection of Carbapenem-resistant Enterobacteriaceae bacteria, especially in patients with VAP (Tuon et al., 2017). High-dose meropenem combined with polymyxin is a limited choice for the treatment of VAP caused by such bacteria, whereas the emergence of polymyxin-resistant strains has resulted in increasingly difficult treatment for CRE infections (Kulengowski et al., 2018).

The mechanisms of resistance of Enterobacteriaceae bacteria to carbapenems are very complex. However, the primary mechanisms include the following types: production of carbapenemases, mainly including KPC and OXA-48; combination of AmpC enzyme or Extended-Spectrum β-Lactamases (ESBLs) with porin loss or reduced expression, resulting in the decreased permeability of the outer membrane; changes in penicillin-binding *proteins* at the drug action site of carbapenem (Cizmeci et al., 2017). The first and second mechanisms are the main resistance mechanisms of drug-resistant strains in China (Wang et al., 2018).

In recent years, given the extensive use of mechanical ventilation in critically ill patients, VAP caused by CRE has increased in China. The high mortality of patients infected by CRE and the lack of effective anti-infection treatment have resulted in additional scientific challenge. Therefore, the study on the prevention and risk factors of VAP may provide significance to its effective control and treatment. We studied the risk factors of infection in VAP patients caused by CRE bacteria and the drug resistance and resistance genes of isolates, providing basis for the protection, and treatment of infected patients.

## Materials and Methods

### Clinical Data

A retrospective study of the prevalence of CRE infection in all VAP patients was conducted in Taihe Hospital from January 2016 to June 2018. Study group cases: The non-repeat CRE strains and clinical data (basic medical history, history of antibiotic use, immune function, and invasive operation, etc.) of patients during the study were collected. 2:1 group case control cases: Patients with the same non-CRE bacterial infection during the same period were selected as the control group, and the same method was used to collect patient data from the case group. The two groups were of similar age and were the infection of CRE bacteria. In accordance with the Declaration of Helsinki, this study was approved by the ethics committee of Taihe Hospital, and written informed consents were obtained from all patients or their families.

### Identification and Drug Sensitivity of Bacteria

The strains were isolated and cultured according to the operating rules of clinical examination in China. The bacterial identification and minimum inhibitory concentration (MIC) detection were carried out by using the DL-96 bacterial determination system. Drug sensitivity test and result assessment were performed according to the MIC method recommended by Clinical and Laboratory Standards Institute (CLSI) in 2015.

### Screening of CRE and Phenotypic Detection of Carbapenemases

The CIM-test utilizes antibiotic susceptibility-testing disks as substrate aliquots. Following 2 h of incubation of a full loop of bacteria with a meropenem disk, the disk is placed on an agar inoculated with *Escherichia coli* ATCC 25922. Enzymatic inactivation will produce no zone, whereas no carbapenemase activity will imply there will be a zone. The criterion for CRE is MIC meropenem or imipenem ≥2 mg/L, and the phenotypes of carbapenemases were detected by mCIM and eCIM experiments as recommended by CLSI 2018.

### The Detection of Resistance Gene

After the bacterial genomic DNA was extracted by boiling method, the resistant genes were amplified. The primers were shown in Table 1. PCR amplification system: ddH2O 20 μl, 5′ and 3′ primer 2 μl, 2 × PCR Master 25 μl, DNA1 μl. Reaction conditions: 94°C denaturation for 5 min, 94°C 30 s, 56°C 30 s, 72°C 45 s with 35 cycles, and extension at 72°C for 5 min; The reaction products were separated electrophoretically on a 2% agarose gel.

**Table 1:** The primers of the resistant genes.

**Gene**	**Forward**	**Reverse**	**Product (bp)**
IMP	TTGACACTCCATTTACTG	GATTGAGAATTAAGCCACTCT	139
KPC	CATTCAAGGGCTTTCTTGCTGC	ACGACGGCATAGTCATTTGC	538
CTX-M-1	CCGAATCTGTTAAATCAGCG	GGTGGTATTGCCTTTCATCC	387
NDM-1	CCAGCTCGCACCGAATGT	GATCAGGCAGCCACCAAAA	475
KPC-2	GCTCATTCAAGGGCTTTCTT	AGGTTCCGGTTTTGTCTCC	504
TEM	ATTTTCGTGTCGCCCTTATTC	CTACGATACGGGAGGGCTTAC	759
SHV	CCGCTTGAGCAAATTAAACA	GCTGGCGATAGTGGATCTTT	214
CTX-M-2	CAGAGCGAGAGCGATAAGCA	AATCTCCGCTGCCGGTTTTA	472
CTX-M-8	CGCTGTTGCTGGGGAG	GTGTTTTTCAGTAATGGGATTGT	293
CTX-M-9	CGATACCGCAGATAATACGC	ATCACCCACAGTCCACGAC	550
OXA	GCTTGATCGCCCTCGATT	GATTTGCTCCGTGGCCGAAA	281
OXA-48	TTGGTGGCATCGATTATCGG	GAGCACTTCTTTTGTGATGGC	743

### Statistical Analysis

The department source, strain distribution, and drug sensitivity of CRE strains were calculated using WHONET 5.6 software. SPSS version 19.0 was employed to analyze the results, Chi^2^ test and univariate analysis were used to analyze the correlation between basic medical history, history of antibiotic use, immune function and invasive operation and CRE infection. Multivariate logistics regression was used to analyze the potential risk factors of CRE infection. *P* < 0.05 was used to indicate statistically significant difference.

## Results

### Distribution of CRE Bacteria

Among the 58 pathogenic bacteria, *Klebsiella pneumoniae* was the most common, followed by *Escherichia coli* and *Enterobacter cloacae* (Table 2). In the distribution of all VAP caused by CRE infection in patients, there were 18 cases in the department of ICU (31.0%), 10 cases in the department of neurosurgery (17.2%),16 cases in the department of respiratory medicine (27.6%) (Table 3)

**Table 2:** The distribution of CRE from VAP patients.

**Abbreviations**	**Bacteria**	***n***	**(%)**
kpn	*Klebsiella pneumoniae*	26	44.8
ecl	*Enterobacter cloacae*	8	13.8
eco	*Escherichia coli*	15	25.8
kox	*Klebsiella oxytoca*	7	12.1
cfr	*Citrobacter freundii*	1	1.7
sma	*Serratia marcescens*	1	1.7
Total		58	100

**Table 3:** Distribution of departments in patients with VAP.

**Department**	***n* (%)**	**kpn**	**E.coil**	**ecl**	**kox**	**cfr**	**sma**
ICU	18 (31%)	9	5	0	3	1	0
Neurosurgery	10 (17.2%)	2	3	3	2	0	0
Respiratory medicine	16 (27.6%)	6	3	4	2	0	1
Urinary surgery	2 (3.4%)	0	2	0	0	0	0
Orthopedics	1 (1.7%)	1	0	0	0	0	0
Pediatric	3 (5.2%)	2	1	0	0	0	0
Oncology	4 (6.9%)	2	1	1	0	0	0
Gerontology	4 (6.9%)	4	0	0	0	0	0
Sum	58						

*kpn, Klebsiella pneumoniae; ecl, Enterobacter cloacae; E. coli, Escherichia coli; kox, Klebsiella oxytoca; cfr, Citrobacter freundii; sma, Serratia marcescens*.

### Drug Sensitive Test Results of CRE

The test strains were resistant to common clinical antibacterial drugs such as cefoperazone/sulbactam, cefuroxime, cefazolin, cefazolin, ampicillin, cefepime, ceficidin, ampicillin/sulbactam. *In vitro* antimicrobial susceptibilities of 58 strains CRE were shown in Table 4.

**Table 4:** *In vitro* antimicrobial susceptibilities of 58 strains CRE.

	**S**		**I**		**R**	
	***n***	**S (%)**	***n***	**I (%)**	***n***	**R (%)**
Chloramphenicol	10	17.2	15	25.9	33	56.9
Minocycline	32	55.2	11	18.9	15	25.9
Trimethoprim/Sulfamethoxazole	25	43.1	1	1.7	32	55.2
Gentamicin	6	10.3	3	5.2	49	84.5
Amikacin	28	48.3	0	0	30	51.7
Imipenem	2	3.4	5	8.6	51	87.9
Meropenem	3	5.2	2	6.4	53	91.4
Polymyxin B	58	100	0	0	0	0
Nitrofurantoin	24	41.4	9	15.5	25	43.1
Levofloxacin	2	3.4	3	5.2	53	91.4

*R, resistant; I, intermediate; S, sensitive*.

### Phenotypic Detection of Carbapenemases in CRE Bacteria

**Figure 1:**
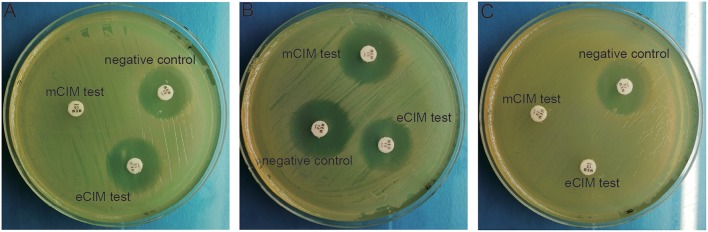
Phenotypic detection of carbapenemases in CRE bacteria. **(A)** mCIM positive and eCIM positive; **(B)** mCIM negative and eCIM negative; **(C)** mCIM positive and eCIM negative.

mCIM and eCIM tests were performed on 58 CRE strains, of which only 23 cases were mCIM positive, 30 cases were mCIM and eCIM positive, 3 cases were negative, and 2 cases could not be identified. Different phenotype detection was shown in Figure 1.

### Amplification Result of Drug Resistance Gene of CRE

**Figure 2:**
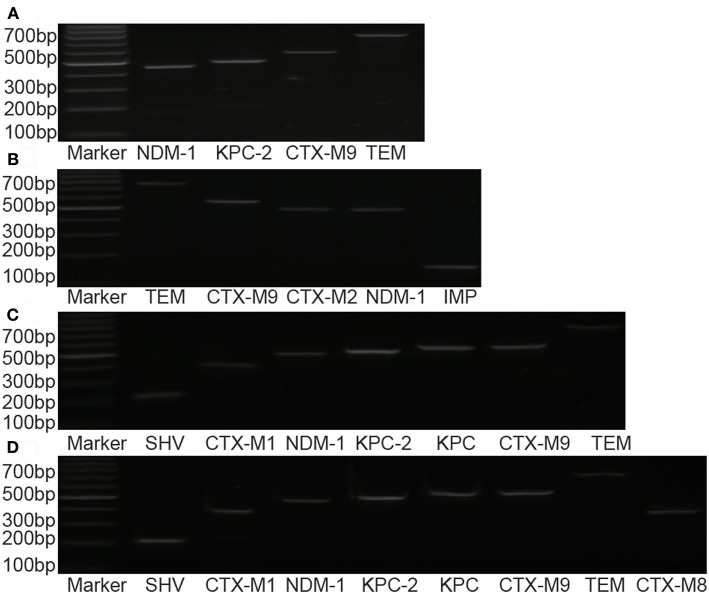
Distribution of drug-resistance genes in different strains. **(A)** *Enterobacter cloacae*; **(B)** *Klebsiella oxytoca*; **(C)** *Escherichia coli*; **(D)** *Klebsiella pneumonia*.

Resistant genes of *Klebsiella pneumoniae, Escherichia coli, Enterobacter cloacae*, and *Klebsiella oxytoca* were detected in 58 strains of pathogenic bacteria, but no drug-resistant genes were detected in *Citrobacter freundii* and *Serratia marcescens* (Figure 2, Table 5).

**Table 5:** Amplification result of drug resistance gene of CRE.

		**IMP**	**KPC**	**CTX-M1**	**NDM-1**	**KPC-2**	**TEM**	**SHV**	**CTX-M2**	**CTX-M8**	**CTX-M9**
kox	7	28.6%	–	–	71.4%	14.3%	57.2%	–	–	–	71.4%
ecl	8	–	75.0%	–	37.5%	50%	50%	25.0%	37.5%	–	75.0%
*E.coli*	15	–	26.7%	86.7%	86.7%	13.3%	80%	26.7%	–	–	40.0%
kpn	26	–	50.0%	42.3%	34.6%	65.4%	57.7%	76.9%	–	11.5%	61.5%

*kpn, Klebsiella pneumoniae; ecl, Enterobacter cloacae; E. coil, Escherichia coli; kox, klebsiella oxytoca*.

### Risk Factors Analysis of CRE Infection

Univariate analysis of the basic medical history, history of antibiotic use, immune function, and invasive operation of the two groups was carried out. The results showed that ICU occupancy time >7 days, cephalosporins containing beta-lactamases inhibitor, carbapenem antibiotics, combined medication, and nerve injury were associated with the occurrence of CRE infection. The mortality and average hospitalization time in CRE infection group were significantly higher than those in control group, and the difference was statistically significant (Table 6).

**Table 6:** Risk factors analysis of CRE infection.

**Variables**	**CRE infection (*n* = 58)**	**Control group (*n* = 116)**	**χ^2^**	**P**	**OR**	**95%CI**
**BASIC INFORMATION**						
Age >60	34	72	1.411	0.235	0.669	0.345~1.298
Gender (male)	23	47	0.334	0.563	0.825	0.430~1.582
ICU >7 d	37	41	9.422	0.002	2.793	1.439~5.421
**BASELINE DISEASES**						
Nerve damage	15	11	6.696	0.009	3.013	1.278~7.101
Liver disease	9	14	0.165	0.685	1.207	0.488~2.988
Diabetes	17	41	1.431	0.232	0.657	0.331~1.307
Tumor	3	11	1.293	0.256	0.471	0.126~1.762
Lung disease	19	24	1.971	0.160	1.665	0.816~3.394
Renal insufficiency	12	33	2.041	0.153	0.577	0.271~1.230
**IMMUNE FUNCTION**						
Granulocytopenia	7	9	0.542	0.462	1.479	0.521~4.203
Splenectomy	1	5	0.947	0.331	0.354	0.040~3.108
Immunosuppressant	3	7	0.133	0.715	0.771	0.192~3.104
**ANTIBIOTIC EXPOSURE**						
Third-generation cephalosporins	27	61	1.812	0.178	0.643	0.337~1.223
Fourth-generation cephalosporins	19	31	0.217	0.641	1.179	0.591~2.349
Quinolones	15	25	0.105	0.746	1.130	0.540~2.367
Aminoglycosides	13	32	1.131	0.287	0.668	0.318~1.405
Enzyme inhibitors	28	34	4.159	0.041	1.977	1.025~3.812
Carbapenems	32	29	12.339	0.0004	3.268	1.671~6.392
Glycopeptides	11	19	0.027	0.869	1.072	0.471~2.411
Antibiotic combination therapy	32	41	4.104	0.043	1.951	1.020~3.732
**INVASIVE OPERATION**						
Catheterization	30	67	2.033	0.154	0.624	0.326~1.193
Venous catheter	23	38	0.231	0.631	1.176	0.608~2.273
**PROGNOSIS**						
Average hospital stay	36 ± 6.6	15 ± 5.2		*P* < 0.01		
Mortality rate	37.2%	11.5%		*P* < 0.01		

### Logistic Regression Analysis of Risk Factors for CRE Infection

Logistic regression analysis showed that ICU occupancy time >7 days (OR = 3.431; 95%CI 1.251~4.152), beta-lactamase inhibitor antibiotics (OR = 3.018; 95%CI 1.102~5.394), carbapenem antibiotics (OR = 5.232; 95%CI 2.579~7.561) were independent risk factor for nosocomial infection of CRE (Table 7).

**Table 7:** Multifactor logistics regression analysis of CRE infection.

**Risk factor**	**B**	**S.E**	**Wals**	**Sig**	**Exp (B)**	**95%CI**
ICU>7d	1.867	0.710	6.914	0.009	6.471	1.609~20.026
Enzyme inhibitors	2.048	0.638	10.296	0.001	7.750	2.219~27.071
Carbapenems	2.213	0.713	9.623	0.002	9.143	2.259~37.01

## Discussion

The infection caused by CRE, especially VAP, has been paid considerable research attention. Although we attempted to control the spread of these resistant bacteria, the effect was negligible, especially as plasmid-mediated carbapenem-resistant strains (KPC-producing) are widely prevalent in the world (Lerner et al., 2013, 2015). The available antimicrobial agents for CRE strains of clinical isolation are limited. Although several combination therapies based on polymyxin, tegacycline, and meropenem are recommended, no clinical evidence is available for their efficacy (Kulengowski et al., 2018; Petty et al., 2018). As an important pathogen of nosocomial infection, CRE can significantly increase mortality, especially in patients with severe diseases (Jovanovic et al., 2015; Landi et al., 2015).

In this study, 58 non-repeat CRE strains were isolated from VAP patients, among which *Klebsiella pneumonia* was the most isolated, followed by *E. coli* (25.8%, 15/58) and *Enterobacter cloacae* (13.8%,8/58), consistent with the prevalence of CRE in China (Yu et al., 2016; Zhang et al., 2017b). ICU is the most common department with cases of CRE infection (31%, 18/58), followed by respiratory medicine (27.6%, 16/58), and neurosurgery (17.2%,10/58), because mechanical ventilation is more common in patients in these departments, and is closely related to the intensity of antibiotic use, invasive operation, and low immunity of patients. Therefore, infection control in these departments may be beneficial to preventing the spread of CRE.

Carbapenemase production is the main drug resistance mechanism of CRE. mCIM and eCIM tests were recommended by CLSI to screen for carbapenemase phenotypes. mCIM was used to detect carbapenemase in Enterobacteriaceae and *Pseudomonas aeruginosa*, whereas eCIM was combined with mCIM to distinguish the Enterobacteriaceae bacteria of metallase- (Ambler B) and serine carbapenemase-producing (Ambler A). The phenotypic results of this study showed that metallase production and serine carbapenemase production are the main drug resistance mechanisms of CRE, which differs from the serine carbapenemase production reported by Tian and Zheng (Zheng et al., 2017; Tian et al., 2018).

The 58 strains of CRE in this study showed multidrug resistance, of which 7 strains showed extensively drug resistance (was only sensitive to polymyxin), Furthermore, no drug-resistant strain was detected. The test strains were resistant to common clinical antibacterial drugs such as cefoperazone/sulbactam, cefuroxime, cefazolin, cefazolin, ampicillin, cefepime, ceficidin, ampicillin/sulbactam. Polycolistin B was the most sensitive antibiotic for CRE in the present study (sensitivity rate 100%). Therefore, polycolistin B was recommended as one of the preferred drugs for treatment of CRE infection. *In vitro* drug sensitivity test, minocycline (sensitivity rate 55.2%), amikacin (sensitivity rate 48.3%), and cotrimoxazole (sensitivity rate 43.1%) were also highly sensitive to CRE, but minocycline and cotrimoxazole acted as antimicrobial agents. The effect of treating severe infection was unclear, and the renal toxicity of amikacin was greater than that of the other drugs. Therefore, the above-mentioned drugs are one of the choices for combination drugs. Nitrofurantoin was also highly sensitive (sensitivity rate 41.4%), but can only be used for urinary tract infections. Avibactam is a new generation of beta lactamase inhibitor for serine carbapenemase enzyme (Ambler A) inhibition, but it lacks metallase (Ambler B) activity (van Duin et al., 2018). Although numerous studies have shown the excellent performance of total/avi cephalosporin treatment of CRE infection, this study offers the class B metal enzymes of CRE strains as the main resistance factor limiting the use of these drugs. Although ceftazidime/avibactam exhibited a good effect in the treatment of CRE infection, the use of this drug was limited by the use of metallase as the main drug resistant enzyme of CRE strain in this study.

By studying the molecular detection and genetic characteristics of CRE, we observed that the gene of carbapenemase and ESBLs (Extended-Spectrum β-Lactamases) were the most important factors involved in mechanisms of drug resistance. A total of 52 strains of bacteria were positive for carbapenemase gene, 30 strains were positive for NDM-1, 24 strains were positive for KPC/ KPC-2, and 2 strains were positive for IMP. Four strains of *Klebsiella pneumonia* were detected with both NDM and KPC, and two positive strains of IMP gene originated from *Klebsiella oxytoca*. Significant differences were observed in the distributions of different resistant genes among these strains. KPC-2 was the most important in *Klebsiella pneumoniae*, whereas NDM-1 was the most important in *Escherichia coli*. Notably, in previous studies in China, a few strains of *Klebsiella pneumonia* with NDM-1 were detected (Hu et al., 2014). However, in our study, 34.6% (9/26) of the strains were detected with NDM-1, and four strains of *Klebsiella pneumoniae* were detected with both NDM and KPC. This finding may be related to local epidemics, suggesting that more attention should be paid to the spread of NDM in this region. The IMP gene was reported in several regions, but was relatively rare in China. In our study, two strains were detected in *Klebsiella oxytoca*. Oxa-48 was more common in Europe but had not been found in our study (Albiger et al., 2015).

ESBLs cannot hydrolyze carbapenem antibiotics, and resistance to carbapenem antibiotics possibly occurs only when accompanied by a membrane pore protein deficiency (Boucher et al., 2016; Bruyère et al., 2016). An interesting phenomenon was that almost all the strains with positive carbapenemase gene presented more than one ESBL gene. The role of ESBL genes in the formation of bacterial resistance had not been clarified, but we noted this phenomenon in other studies in the past (Zhang et al., 2017a).

The selection pressure of antibiotics is an important factor in producing multidrug resistance of bacteria. The selection pressure of carbapenem antibiotics on Enterobacteriaceae was beneficial to the screening of carbapenem-producing strains, which led to the mass production of CRE strains (Datta et al., 2015; Mariappan et al., 2017). The reasons for the susceptibility to CRE in the use of broad-spectrum antibiotics with β-lactamase inhibitors to CRE include the screening of antibiotics and the induction of β-lactamase inhibitors. The combination of antibiotics was a risk factor for CRE infection in other studies but not in our research. This condition suggests that the main factors leading to higher CRE infection may be the use of broad-spectrum antibiotics with β-lactamase inhibitors and carbapenem antibiotics, whereas the combination of antibiotics serves as a confounding factor.

ICU occupancy time>7 days is another important risk factor for CRE infection. A longer duration of stay of patients in the ICU indicates a higher chance of nosocomial infection. Moreover, more invasive operations lead to higher risk of exposure to drug-resistant bacteria, which show increasing drug resistance of bacteria. Therefore, we should select antibiotics according to drug sensitivity test results to reduce the production of CRE-resistant strains. We also noted that the mortality of patients with CRE infection was significantly higher than that of the control group, and the average hospitalization time was longer than that of the control group. Other studies had shown that the hospitalization cost of CRE infection also significantly increased. This result was attributed to the limited anti-infection treatment of CRE and the high number of drugs that must be combined. Furthermore, the infection of patients was often accompanied with serious diseases (You et al., 2018).

## Conclusion

In conclusion, the CRE strains were isolated from VAP had strong drug resistance and high mortality in infected patients. Strengthening the management and application of clinical antimicrobial agents, isolating and protecting patients with ICU, and standardizing clinical operation were effective methods to control and prevent the spread of CRE.

## Data Availability

The datasets generated for this study are available on request to the corresponding author.

## Author Contributions

ZZ participated in the research design. BG, XL, YZ, and GB conducted the experiments. FY and WC performed the data analysis. ZZ and BG wrote the manuscript.

### Conflict of Interest Statement

The authors declare that the research was conducted in the absence of any commercial or financial relationships that could be construed as a potential conflict of interest.
